# ItaLynch: an ongoing Italian study to evaluate the feasibility of mainstreaming the diagnosis of Lynch syndrome in colorectal cancer patients

**DOI:** 10.1016/j.esmogo.2024.100044

**Published:** 2024-03-05

**Authors:** A. Puccini, F. Grillo, M. Fassan, S. Lonardi, M. Genuardi, R. Cannizzaro, G.M. Cavestro, F. Marmorino, V. Conca, L. Salvatore, F. Bergamo, F. Tosi, F. Morano, V. Daprà, C. Molica, D. Barana, A. Guglielmi, C. Signorelli, M. D’Amico, F. Zoratto, D. Iacono, A. Morabito, G. Martini, A. Fabbroncini, M. Duro, G. Bruera, A. Auriemma, B. Bonanni, A. Percesepe, M. Dono, L. Battistuzzi, R. Labianca, L. Boni, S. Sciallero, ItaLynch Working Group

**Affiliations:** 1 Oncologia Medica 1, IRCCS Ospedale Policlinico San Martino, Genova; 2 Medical Oncology and Hematology Unit, IRCCS Humanitas Research Hospital, Humanitas Cancer Center, Rozzano, Milan; 3 Anatomic Pathology Unit, IRCCS Ospedale Policlinico San Martino, Genoa; 4 Department of Surgical and Integrated Diagnostic Sciences (DISC), University of Genoa, Genoa; 5 Department of Medicine (DIMED), Surgical Pathology Unit, University of Padua, Padua; 6 Veneto Institute of Oncology IOV-IRCCS, Padua; 7 Department of Oncology, Veneto Institute of Oncology IOV-IRCCS, Padua; 8 UOC Genetica Medica, Dipartimento di Scienze di Laboratorio e Infettivologiche, Fondazione Policlinico Universitario A. Gemelli IRCCS, Rome; 9 Sezione di Medicina Genomica, Dipartimento di Scienze della Vita e Sanità Pubblica, Università Cattolica del Sacro Cuore, Rome; 10 Department of Medical, Surgical, and Health Sciences, University of Trieste, Trieste; 11 Oncological Gastroenterology, Centro di Riferimento Oncologico di Aviano (CRO) IRCCS, Aviano; 12 IRCCS, Italy Gastroenterology and Gastrointestinal Endoscopy Unit, Vita-Salute San Raffaele University, IRCCS San Raffaele Scientific Institute, Milan; 13 Unit of Medical Oncology 2, University Hospital of Pisa, Pisa; 14 Department of Translational Research and New Technologies in Medicine and Surgery, University of Pisa, Pisa; 15 Oncologia Medica, Comprehensive Cancer Center, Fondazione Policlinico Universitario Agostino Gemelli IRCCS, Roma; 16 Oncologia Medica, Università Cattolica del Sacro Cuore, Roma; 17 Department of Hematology, Oncology, and Molecular Medicine, Grande Ospedale Metropolitano Niguarda, Milan; 18 Fondazione IRCCS Istituto Nazionale dei Tumori di Milano, Milan; 19 Medical Oncology Unit, S. Maria della Misericordia Hospital, Perugia; 20 Oncology Unit, Local Health and Social Care Unit, ULSS8 Berica, Montecchio Maggiore, Vicenza; 21 Oncology Department, University Hospital of Trieste, Trieste; 22 Medical Oncology Unit, Department of Oncology and Hematology, Central Hospital of Belcolle, Strada Sammartinese Snc, Viterbo; 23 E.O. Ospedali Galliera, Genoa; 24 Oncology Unit, S.M. Goretti Hospital, Latina; 25 Department of Oncology, ASUFC University Hospital of Udine, Udine; 26 Department of Oncology, AULSS6 Euganea, Cittadella Camposampiero; 27 Medical Oncology, Department of Precision Medicine, University of Campania ‘Luigi Vanvitelli’, Naples; 28 Oncology Unit, Ospedale del Mare, Naples; 29 Ospedale Valduce - Como, Como; 30 Oncology Territorial Care, S. Salvatore Hospital, ASL1 Abruzzo, University of L’Aquila, L’Aquila; 31 Section of Oncology, Department of Medicine, University of Verona, Azienda Ospedaliera Universitaria Integrata (AOUI) di Verona, Verona; 32 IEO, European Institute of Oncology IRCCS, Division of Cancer Prevention and Genetics, Milan; 33 Medical Genetics, Department of Medicine and Surgery, University of Parma, Parma; 34 Molecular Diagnostic Unit, IRCCS Ospedale Policlinico San Martino, Genoa; 35 Medical Oncology Unit 2, IRCCS Ospedale Policlinico San Martino, Genoa; 36 Cancer Center ASST Papa Giovanni XXIII, Bergamo; 37 Clinical Epidemiology Unit, IRCCS Ospedale Policlinico San Martino, Genova, Italy

**Keywords:** DNA mismatch repair deficiency, colorectal cancer, Lynch syndrome, universal screening, reflex testing

## Abstract

**Background:**

International guidelines recommend universal screening for Lynch syndrome (LS) through somatic DNA mismatch repair deficiency (dMMR) testing in all colorectal cancers (CRCs). However, LS remains largely underdiagnosed. Mainstreaming LS diagnosis through oncologist-driven genetic testing could increase detection rates, thus extending the benefits of precision prevention to patients with LS and their families. We aim to evaluate the feasibility of the mainstreaming diagnostic algorithm for LS.

**Patients and methods:**

ItaLynch is an ongoing, prospective, observational, multicenter, multidisciplinary, Italian study in patients with dMMR CRC. Being descriptive in nature, it does not attempt to test any specific, *a priori*, hypothesis. Patients with dMMR CRC are selected by universal screening by immunohistochemistry (IHC). In *MLH1*-deficient patients, reflex testing for *BRAF*^*V600E*^ and, when appropriate, for *MLH1* promoter hypermethylation is carried out. For all dMMR CRC, a ‘Lynch Alert’ is added to the pathology report: positive when a patient is at high risk for LS, due to reflex testing results or to loss of non-MLH1 proteins. Conversely, a ‘Lynch Alert’ is negative when the patient is likely to be a nonhereditary case (i.e. MLH1 loss and *BRAF*^*V600E*^ or *MLH1* promoter hypermethylation). In patients with a positive ‘Lynch Alert’, after providing a brief explanation about the risks and benefits of genetic testing, the oncologist asks patients for their consent to mainstream genetic testing. Thus a blood sample is drawn for constitutional variants of the MMR genes. Carriers of a germline variant are then referred to post-test genetic counseling. Referral to clinical genetic services is also advised for patients with clinical suspect criteria.

## Highlights

• This is a prospective, observational, multicenter, multidisciplinary, Italian study.
• We investigate the feasibility of a new diagnostic algorithm for LS.
• A ‘Lynch alert’ in the pathology report is used as a clear warning to clinicians.
• We propose a shift to an oncologist-led mainstreaming genetic testing.
• This study encourages the establishment of multidisciplinary workgroups.

## Background

### Colorectal cancer, DNA mismatch repair deficiency, and Lynch syndrome

Colorectal cancer (CRC) is the third most common type of cancer diagnosed and the second leading cause of cancer death worldwide.^1^ In Italy, CRC is the second most frequently diagnosed and the second leading cause of cancer death.^2^

Overall, ∼15% of all patients with CRC (stage I-IV) have DNA mismatch repair deficiency (dMMR), which results in high microsatellite instability (MSI-H).^3^ Of note, the prevalence of dMMR varies according to stage and location. Indeed, among patients with CRC, dMMR prevalence is higher in early stages and decreased in advanced disease (20% in stage I-II, 12% in stage III, and 4% in stage IV). Moreover, the prevalence of dMMR in rectal cancer is ∼3%.^4^ Among patients with dMMR CRC, ∼20% are affected by Lynch syndrome (LS).^5^ This estimate suggests that the prevalence of LS among all patients with CRC is ∼3%.

LS is a common inherited cancer predisposition disorder estimated to affect 1 in 279 individuals worldwide.^6^ The syndrome is caused by germline pathogenic variants in DNA mismatch repair (MMR) genes (*MLH1, MSH2, MSH6,* or *PMS2*) or the *EPCAM* gene.^7^ In LS, lifetime risks are highest for CRC and endometrial cancers; the risks of ovarian, stomach, small bowel, urothelial, pancreaticobiliary, and brain cancers are also increased compared with the general population.^5^^,^^8^

#### Why diagnosing LS is important

Patients with LS-associated CRC seem to have a better prognosis compared with patients with sporadic CRC.^9^^,^^10^ Furthermore, patients with LS require personalized treatment and benefit from enrollment in targeted surveillance protocols for metachronous primary CRCs and other syndrome-associated cancers.^11^ Identifying the at-risk relatives of patients with LS and enrolling them in surveillance programs also leads to early diagnosis of LS-associated cancers, reducing mortality by ∼60%.^12^

#### Diagnosing LS

The clinical diagnosis of LS used to be based on clinical features, including family history of cancer, and pathological findings (Amsterdam criteria 1990 and Bethesda criteria 1997).^13^^,^^14^ However, these diagnostic criteria fail to recognize LS in ∼25% of cases,^15^^,^^16^ likely due to their complexity and to the fact that accurate family histories are seldom available. Therefore universal screening of LS through the identification of MMR deficiency in the tumor tissue of all CRC cases has been proposed as an alternative diagnostic approach since 2008.^17^

### The universal screening algorithm for LS

The universal screening for LS^18^, ^19^, ^20^ includes IHC test to evaluate MMR protein expression loss or a molecular test to assess MSI.^17^^,^^21^ Multiple societies currently recommend testing for patients with newly diagnosed CRC, and this is now the standard of care.^22^ Overall, IHC is to be preferred over the MSI test because it is cheaper and more widely available; it can also predict good response to treatment with immune checkpoint inhibitors in patients with advanced cancer.^23^^,^^24^ Finally, it is more informative than the MSI test, as it is able to distinguish which of the four MMR proteins is involved, thereby guiding reflex testing, genetic counseling, and genetic testing.^25^

### Reflex testing

Genetic counseling and testing are always recommended for patients with CRC with MSH2, MSH6, or PMS2 protein expression loss, given that they are likely to be affected by LS.^26^ CRCs with MLH1 protein expression loss, who account for the vast majority of patients with dMMR CRC, are instead more frequently nonhereditary, due to somatic epigenetic inactivation of the *MLH1* gene. As the presence of somatic *BRAF*^*V600E*^ mutation and/or hypermethylation of the *MLH1* promoter are indicative of a very low likelihood of LS, reflex tumor testing has been implemented to better select patients with MLH1 loss for genetic counseling and testing.^27^

Several strategies have been investigated for cost-effectiveness by the National Institute for Health and Care Excellence (NICE), which suggests that strategies beginning with IHC for MMR proteins are more cost-effective than those beginning with MSI testing, and that overall, reflex testing conducted as described below appears to be the most cost-effective approach^21^:

The IHC four-panel test for MLH1, MSH2, MSH6, and PMS2, then:

• genetic testing for abnormal MSH2, MSH6, or PMS2 IHC results, or
• *BRAF*^*V600E*^ testing for an abnormal MLH1 IHC result; if negative, then *MLH1* promoter hypermethylation testing is carried out; if the *MLH1* promoter is not hypermethylated, genetic testing is carried out.

### Oncologist-led ‘mainstream’ genetic testing

The identification of patients whose cancer is associated with an underlying predisposition syndrome allows for improved understanding and management of their own and their relatives’ future cancer risk. Increasingly, it also has an impact on their treatment. Considering the potential benefits for both treatment and prevention, National Comprehensive Cancer Network (NCCN) guidelines have endorsed germline genetic testing for all patients with certain cancer types (epithelial ovarian cancer, exocrine metastatic pancreatic cancer, and high-grade/metastatic prostate cancer), regardless of age or personal/family history of cancer.^28^

However, ensuring appropriate and equitable access to genetic counseling and testing remains a challenge, as increased referral volumes have translated into over-burdened genetic services and longer waiting times for access to genetic counseling. An alternative model, named ‘mainstreaming cancer genetics’, was therefore developed^29^, ^30^, ^31^, ^32^ and successfully trialed.^33^ It has been shown that it can improve access to hereditary cancer genetic testing in patients with breast and ovarian cancer by shifting genetic testing from genetic services to oncology clinics, enabling oncologists to discuss and initiate genetic testing with selected patients with cancer.

Recently, a systematic review has shown that mainstream genetic testing seems feasible in daily practice, with no insurmountable barriers. A structured pathway with a training procedure is desirable, as well as close collaboration between genetics and other clinical departments.^34^

To maximize availability, utility, and equity of access to genetic testing for LS, all patients found to be at risk for LS by reflex testing could be offered genetic testing by oncologists.

Based on this background, the overall goal of our study is to evaluate the feasibility and performance of an oncologist-driven diagnostic algorithm for LS (mainstreaming) in patients with CRC.

### Study rationale

In the traditional diagnostic pathway for LS, genetic counseling and testing are always recommended for patients with CRC in whom loss of expression of one of the proteins encoded by the *MSH2, MSH6,* or *PMS2* genes is detected. Identifying which patients with MLH1 protein expression loss (who account for ∼80% of all dMMR cases) should be offered genetic counseling and genetic testing is not straightforward, as protein expression loss in most of these patients is not caused by a germline pathogenic variant,^15^ but rather by somatic epigenetic methylation of the *MLH1* promoter. Moreover, CRCs in LS carriers do not usually display a *BRAF*^*V600E*^ mutation.^17^^,^^21^ Therefore, in case of MLH1 loss, genetic counseling and genetic testing are usually recommended only for patients with *BRAF* wild-type with no hypermethylation of the *MLH1* promoter.

International guidelines (NCCN) do not detail the role of different health care professionals in the decisional algorithm leading to a diagnosis of LS. *BRAF*^*V600E*^ testing carried out routinely by the pathologist in all *MLH1*-deficient tumors has been found to determine a 40% reduction in referrals to genetic clinics, improving the appropriateness of referrals, leading to time and cost savings, and sparing unnecessary alarm to patients.^35^ Moreover, the evaluation of *MLH1* hypermethylation has been reported to potentially spare an additional 60% of patients with *BRAF* wild-type the need for genetic counseling.^36^

Preliminary data from our study conducted on ∼1500 patients at IRCCS Policlinico San Martino in Genoa showed that the rate of referral of patients with dMMR CRC to pretest genetic counseling by oncologists is low (only 20%). Moreover, we observed that if pretest counseling had been offered only to the patients with *BRAF* wild-type with MLH1 loss, 57% of the pretest counseling sessions conducted could have been avoided, increasing the genetic counseling yield in terms of LS diagnosis by 75%.^37^^,^^38^

Taken together, these findings suggest that incorporating genetic testing in an oncologist-driven diagnostic algorithm for LS could increase diagnostic rates, offering the benefits of precision medicine and a streamlined pathway of care to patients with LS and their families.^39^^,^^40^

## Patients and methods

### Overall study design

This is a prospective, observational, multicenter, Italian study of patients diagnosed with dMMR/MSI-H CRC. The study is descriptive in nature and does not attempt to test any specific *a priori* hypotheses. Patients are recruited from participating sites from all over Italy.

Our multidisciplinary project involves oncologists, pathologists, molecular biologists, gastroenterologists, and geneticists to make LS diagnosis as universal and simple as possible in all participating centers. We have established a nation-wide network across Italian centers, involving those able to carry out IHC analysis of MMR protein in all patients with stage I-IV CRC (LS universal screening) and that already have well-established links with medical genetics services.

Patients in whom CRC tumor samples are found to be dMMR/MSI-H at each participating site are enrolled in the study according to the inclusion/exclusion criteria, after informed consent. *BRAF*^*V600E*^ mutation and *MLH1* hypermethylation are evaluated in MLH1-deficient patients at all centers, in accordance with the reflex testing model.

For all patients with dMMR/MSI-H CRC, a ‘Lynch Alert’ is added to the pathology report, warning oncologists and/or gastroenterologists about the patient’s risk of being affected by LS or not. We define a ‘Lynch alert’ as positive when a patient with dMMR/MSI-H is at high risk for LS, due to the results of reflex testing or to loss of non-MLH1 proteins. Conversely, a negative ‘Lynch alert’ is added to the pathology report when the patient is likely to be a nonhereditary dMMR/MSI-H case (i.e. MLH1 loss and *BRAF*^*V600E*^ mutation or *MLH1* promoter hypermethylation).

After providing a brief explanation about the risks and benefits of genetic testing, possible results, and their implications, oncologists and/or gastroenterologists ask patients with a positive ‘Lynch Alert’ for their consent to mainstream genetic testing. If the patient consents, a blood sample is drawn to search for constitutive variants of the *MLH1, MSH2, MSH6*, and *PMS2* genes. Standard site-specific procedures (e.g. informed consent forms for routine genetic testing and transfer of blood sample to testing laboratory) are employed by all centers. Blood samples are analyzed by local medical genetics laboratories, which return the results to treating oncologists and/or gastroenterologists. This avoids referring all patients with dMMR for pretest genetic counseling. Participants in whom a pathogenic variant of one of the MMR genes is detected are referred for post-test genetic counseling.

Every patient found to be:

• MMR proficient or
• not eligible for genetic testing (dMMR with an MLH1 deficiency and a *BRAF*^*V600E*^ mutation or MLH1 hypermethylation—negative ‘Lynch Alert’) or
• with a negative genetic test result

is considered unlikely to be affected by LS. Nevertheless, to rule out the presence of a hereditary cancer syndrome, oncologists and/or gastroenterologists administer a validated family history questionnaire to all participants. Participants whose questionnaire results suggest the presence of a hereditary cancer syndrome are referred to genetic counseling. Participants who wish to receive more in-depth information about their genetic risk are also referred to genetic counseling.

In addition, all ItaLynch participants found to harbor variants of uncertain significance are referred for post-test counseling. In addition, a parallel study on variants of uncertain significance is being carried out by the geneticists involved in the ItaLynch study.

Most oncology units included in this project participated in the TOSCA Trial, sponsored and coordinated by the Italian Group for the study of gastrointestinal cancers (GISCAD), the first trial to be conducted and completed among the international IDEA trials on adjuvant colon cancer treatment.^41^^,^^42^ The results of these studies led to a paradigm shift in the treatment of patients with early-stage colon cancer worldwide. Such an accomplishment was made possible thanks to the close, successful cooperation among groups from all over Italy and coordination by the GISCAD group which allowed us to enroll ∼3700 patients with colon cancer.

The start day of the study was 1 March 2021, which is the day the study was approved by the Ethics Committee of the Coordinating Center in Genoa, Italy. The project will continue to prospectively enroll patients in for 48 months overall, so the planned end date will be in March 2025.

### Study objectives

The primary objectives of the study are as follows:

• To estimate the frequency of LS among patients with dMMR CRC.
• To evaluate the feasibility of the mainstreaming diagnostic algorithm for LS.
• To estimate the extent to which the mainstreaming approach reduces the workload of specialist genetics services compared with the traditional genetic counseling model.
• To measure patients’, oncologists’, and geneticists’ satisfaction with the mainstreaming approach.

### Endpoints

• Proportion of LS diagnosis among patients with dMMR/MSI-H enrolled in the study.
• Proportion of:
  • BRAF^V600E^ testing among MLH1-deficient cases.
  • *MLH1* promoter hypermethylation testing among BRAF wild-type cases.
  • ‘Lynch Alert’ addenda in the histology report of all dMMR/MSI-H cases.
  • Oncologist/gastroenterologist-led mainstream genetic testing among participants with a positive ‘Lynch Alert’.
  • Participants agreeing to have clinical genetic testing among those invited within the oncologist/gastroenterologist-led mainstream genetic testing process.
  • Oncologist/gastroenterologist referring participants with a positive genetic test result for post-test counseling.
  • Participants agreeing to post-test genetic counseling.
  • Participants presenting to post-test genetic counseling.
• Ratio of:
  • Post-test genetic counseling sessions following the mainstream diagnostic process compared with the estimated number of post-test genetic counseling sessions according to the classical diagnostic model.
  • Patients requesting referral to pretest genetic counseling among those offered genetic testing by oncologists/gastroenterologists.
  • Patients requesting referral to post-test genetic counseling among those with negative genetic testing.
• Satisfaction of:
  • Patients after mainstream pretest counseling, using the Oncogenetic Counseling Elements Questionnaire.
  • Patients after genetic testing, using the Modified Royal Marsden Patient Satisfaction Questionnaire.
  • Oncologists, using the Oncologist Satisfaction Survey.
  • Geneticists, using the Genetic Counselor Satisfaction Survey.

### Eligibility

#### Inclusion criteria

• Histologically confirmed diagnosis of stage I-IV colorectal adenocarcinoma with dMMR.
• Signed informed consent to participate to the study.

Note: The signed informed consent required for clinical genetic testing and blood sampling collection is needed only for patients found to be eligible for this procedure during the study.

#### Exclusion criteria

• Significant psychiatric or clinical impairment compromising consent to the study.

Formal registration of patients into the study is managed through the web-based electronic system developed by the Clinical Epidemiology Unit, IRCCS Ospedale Policlinico San Martino, Genova, Italy and available at https://eclintrials.org/ect.

#### Flowchart

Oncologist/gastroenterologists select patients with dMMR/MSI-H CRC referred to each participating site per the inclusion/exclusion criteria.

**Figure 1:**
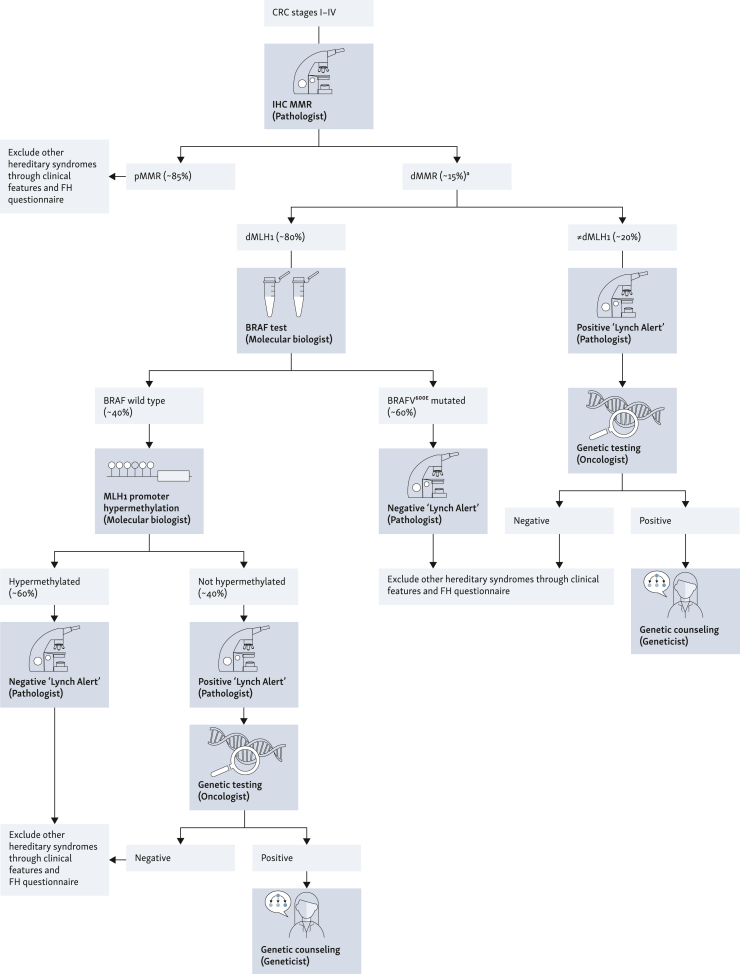
**Diagnostic algorithm for Lynch syndrome diagnosis through the ‘mainstream’ process.**^a^At this time point, patients with dMMR CRC are selected by oncologists/gastroenterologists according to the inclusion/exclusion criteria at each participating site. Selected patients are invited to give their informed consent to participate in the study. CRC, colorectal cancer; dMMR, DNA mismatch repair deficiency; FH, family history; pMMR, proficient DNA mismatch repair.

Patients undergo the following diagnostic algorithm (Figure 1):

- Immunohistochemical diagnosis of dMMR CRC cases
- Oncologist/gastroenterologist-led consent and blood drawing for clinical genetic testing in all patients with loss of MSH2, MSH6, and PMS2 expression

Given the complexity of the selection criteria for genetic testing, oncologists are supported by:

1. Implementation of reflex testing, which is the automatic evaluation of *BRAF*^*V600E*^ mutation and *MLH1* promoter hypermethylation in all dMMR cases due to MLH1 loss.
2. For all patients with dMMR/MSI-H CRC, a ‘Lynch Alert’ added to the pathology report, warning Oncologist/gastroenterologists about the patient’s risk of being affected by LS or not.

The blood sample drawn in the oncology or gastroenterology department is sent to the laboratory of the cooperating specialist genetics service. If the test result is positive, the genetics laboratory communicates it to the oncologist/gastroenterologist, who then communicates it to the participant and refers them for post-test genetic counseling.

Patients participating in the study are followed from the date of enrollment, disclosure of genetic test results, after genetic test counseling, completion of the satisfaction survey, or death.

An electronic case report form has been developed to collect information on the primary variables of interest (i.e. reflex testing and Lynch alert completion, genetic testing, and genetic counseling turnaround time), participant and disease characteristics, medical history, cancer family history, and outcome of genetic counseling. The electronic case report forms are managed through the same web-based electronic system used for the registration of patients.

In addition, validated questionnaires^33^ are used to evaluate patients’, oncologists’, and geneticists’ satisfaction. Specifically, patient satisfaction is evaluated two times:

1. After mainstream pretest counseling, before genetic testing, using the Oncogenetic Counseling Elements Questionnaire: this questionnaire asks patients whether key points related to testing (purpose, implications, accuracy) have been discussed and then asks them to provide an overall rating of how satisfied they are with the counseling process.
2. After genetic testing, using the Modified Royal Marsden Patient Satisfaction Questionnaire: this questionnaire asks patients if they are satisfied with the testing process, and specifically whether they are happy they had the test during one of their oncology appointments.

Oncologists evaluate the novel testing pathway using the Oncologist Satisfaction Survey (14 questions), which investigates clinicians’ opinions on offering genetic testing for LS to patients and on how the process works.

Geneticists evaluate the novel pathway using the Genetic Counselor Satisfaction Survey (seven questions), which explores genetics professionals’ view on other health care professionals carrying out genetic testing and on whether patients seen post-test seem to have received accurate information before testing.

#### Statistical methods

For descriptive purpose all proportions will be presented with corresponding 95% confidence intervals. Because of the descriptive intent of the study, a formal statistical hypothesis to be tested is not identified. Consequently, the calculation of the required sample size has not been carried out. However, it has been estimated that during the 4 years of accrual a total of 1140 patients with dMMR/MSI-H CRC/year will be registered into the study from all participating clinical sites. According to this estimate, the expected number of patients with an LS diagnosis is ∼228/year (20 of all dMMR/MSI-H CRC cases; see the ‘Expected Results’ section for more details).

## Expected results

From a preliminary survey among over 130 Italian centers where gastrointestinal cancers are treated, 43 have shown interest in participating in the project (Supplementary Table S1, available at https://doi.org/10.1016/j.esmogo.2024.100044). The patients diagnosed with CRC at all centers are estimated to be ∼7600 per year, and we expect an ∼15% proportion of dMMR cases (*N* = 1140 per year). Among these patients with dMMR, ∼80% will present MLH1 loss (versus 20% of cases with MSH2, MSH6, and PMS2 loss), 40% of whom will have wild-type *BRAF*^*V600E*^. Of these, ∼40% will not present *MLH1* promoter hypermethylation and thus will be eligible for genetic testing, along with the patients with loss of MSH2, MSH6, and PMS2.^36^ Therefore, overall, one-third of patients with dMMR will be offered oncologist- or gastroenterologist-driven genetic testing (∼5% of all patients with CRC). Around half of these patients are expected to have a positive genetic test result and will be referred for post-test genetic counseling (∼2%-3% of all patients with CRC). Genetic counseling will then provide information about the syndrome and implications in terms of surveillance and other preventive measures for the patient and his/her relatives. Considering the prevalence of 2%-3% of LS in all patients with CRC, we expect to find ∼150-228 cases per year, based on full active enrollment by all centers and 100% compliance with the algorithm.

### Ethical considerations

The process of identifying the subset of patients with CRC who are affected by LS through an oncologist/gastroenterologist-led diagnostic process may be associated with ethical issues having to do, for instance, with informed consent. An ethics advisor with specific expertise in cancer genetics (LB) participates in the project to analyze the issues at hand and propose ethically appropriate approaches, including preparing consent materials.

The study was first approved by the Ethics Committee of the Coordinating Center—Comitato Etico Regionale (CER), Liguria, Ospedale Policlinico San Martino, Genoa, Italy (N. Registro CER Liguria: 102/2021 - DB id 11075).

The study was then approved by the Ethics Committees of all the participating centers, which are listed in the Supplementary Table S1, available at https://doi.org/10.1016/j.esmogo.2024.100044.

#### Patient and public involvement

Patients or the public were not involved in the design, or conduct, or reporting, or dissemination plans of our research.

## Discussion

Although universal screening for LS was first suggested in 2008-2009,^15^^,^^43^ this syndrome is still largely underdiagnosed. It has been estimated that up to 98% of individuals affected by LS have yet to be identified.^44^

Identifying patients with CRC and families affected by LS will benefit both the patients and their at-risk relatives. The patients will undergo specific surveillance and follow-up for the early diagnosis of subsequent cancers. The at-risk relatives who test positive for LS will be offered targeted surveillance for the early diagnosis of tumors and have the option to choose prophylactic surgery, according to the standard of care after post-test genetic counseling.

Universal screening and reflex testing have been shown to be cost-effective^21^ and have been classified by the Center for Disease Control (CDC) as one of the highest-tier programs in terms of reduction of mortality and morbidity and improvement of quality of life. Besides, universal screening and reflex testing are recommended by several international scientific societies, such as the American Society of Clinical Oncology (ASCO), the European Society for Medical Oncology (ESMO), the NICE, the NCCN, and the Healthy People 2020 Initiative.

Demonstrating the feasibility and efficacy of our oncologist/gastroenterologist-led diagnostic algorithm in high-volume cancer centers will make it possible to extend the program to all Italian cancer centers.

‘Mainstreaming’ is an innovative model of LS diagnosis. In addition to the benefits granted by the universal screening, it will have a strong positive impact in terms of improving the diagnosis of LS, cost-efficiency, and reduction of the workload and waiting list in the genetics units. In addition, it will improve clinicians’ compliance with international guidelines.

Two recent papers issued a ‘call to action’ to all health professionals to develop and spread large-scale precision prevention programs to identify individuals with LS and other clinically relevant hereditary syndromes, given their impact on the population.^45^^,^^46^

We fully support this proposal, and our project is an attempt to develop such a program.

Precision oncology is dramatically reshaping cancer care, as molecular profiling is necessary for clinical decision making in several tumor types,^47^ although the interpretation of molecular data remains a challenge.^48^ Potential consequences of testing are the identification of variants that can have implications other than treatment choice, such as inherited alterations that require confirmatory germline DNA testing, with potential impacts on both the patient and their families.^49^ All these issues raise ethical and practical questions about whether and how these results should be reported.^50^

For these reasons and due to the importance of these aspects, the ASCO recently published a provisional clinical opinion which addresses the appropriate use of tumor genomic testing in patients with metastatic or advanced solid tumors, and a new guideline on germline genetic testing, including genetic counseling, is expected in Spring 2023.^51^ Germline testing for genetic alterations linked to approved therapies should be carried out in patients with metastatic solid tumors, and it should not be limited by family history-based or clinical criteria used for familial risk assessment.^52^ Patients who are found to carry pathogenic or likely pathogenic variants should then be referred to genetic counseling. More importantly, when carrying out tumor-only testing, it is recommended that clinicians should refer patients for genetic counseling and further germline testing when pathogenic or likely pathogenic alterations are found in genes associated with germline alterations.^51^^,^^53^ In addition, the ESMO Expert Consensus Statements on Cancer Survivorship state that patients with hereditary syndromes benefit from informed and tailored screening strategies in follow-up.^54^

The ItaLynch study embraces these principles. It aims to increase LS diagnosis in all patients with dMMR/MSI-H CRC, by demonstrating the feasibility of the mainstream approach. In addition, its goal is to increase knowledge and raise awareness about this hereditary condition among all stakeholders. As mentioned earlier, although universal screening for dMMR/MSI-H has been proposed for more than a decade now, it is not widely implemented among pathology units and oncologists’ adherence to national and international guidelines to identify LS carriers has been poor.^55^

Furthermore, oncologists are increasingly requesting this test. This is due to the universal screening Food and Drug Administration (FDA)’s first tissue/site-agnostic approval of pembrolizumab for patients with unresectable or metastatic, dMMR/MSI-H solid tumors that have progressed following prior treatment in 2017,^56^ as well as to the extraordinary results and the availability of immunotherapy for patients with dMMR/MSI-H CRC.^57^^,^^58^ Despite such wide use of dMMR/MSI-H testing, oncologists often overlook its potential hereditary implications.

In our view, dMMR/MSI-H testing is the one of the best examples of a universal somatic test that is essential for both therapy and genetic counseling/testing guidance. This is due to the well-established, clear benefit of surveillance programs in both patients and in at-risk relatives, as well as the potential personalized approach to lifelong gene-specific management for people with LS.^59^

The broad implementation of universal screening, reflex testing and mainstreaming, upon demonstration of its feasibility through the ItaLynch study, will enable the most accurate selection of patients with dMMR/MSI-H CRC who need genetic testing and counseling. This will increase LS diagnosis, reducing the workload of specialist genetics services and empowering patients in shared decision making.
