# Supplementary material for: ItaLynch: an ongoing Italian study to evaluate the feasibility of mainstreaming the diagnosis of Lynch syndrome in colorectal cancer patients
Source: ESMO Gastrointest Oncol. 2024 Mar 5;3:100044. doi: 10.1016/j.esmogo.2024.100044 (PMC12836580; doi:10.1016/j.esmogo.2024.100044)
Supplement: Supplementary Table 1 [file mmc1.pdf]

Supplementary Table 1. List of participating centers

	**Center**	**EC Evaluation**	**EC approval**	**CRF activated**
**1**	**PI - Azienda Ospedaliera Universitaria Pisana**	**Yes**	**Yes**	**Yes**
**2**	**GE - IRCCS S. Martino**	**Yes**	**Yes**	**Yes**
**3**	**PD - IOV- IRCCS**	**Yes**	**Yes**	**Yes**
**4**	**RM - Fondazione Policlinico Gemelli**	**Yes**	**Yes**	**Yes**
**5**	**MI - ASST Grande Ospedale Metropolitano Niguarda**	**Yes**	**Yes**	**Yes**
**6**	**MI - Fondazione IRCCS Istutito Nazionale Tumori (INT)**	**Yes**	**Yes**	**Yes**
**7**	**MI - ICH Humanitas**	**Yes**	**Yes**	**Yes**
**8**	**PG - Azienda Ospedaliero Universitaria Perugia**	**Yes**	**Yes**	**Yes**
**9**	**VI - Azienda ULSS 8 Berica - Montecchio Maggiore**	**Yes**	**Yes**	**Yes**
**10**	**VT - ASL Viterbo**	**Yes**	**Yes**	**Yes**
**11**	**TS - ASUITS Trieste**	**Yes**	**Yes**	**Yes**
**12**	**GE - Ospedale Galliera UOC di Oncologia**	**Yes**	**Yes**	**Yes**
**13**	**LT - UOC Oncologia Medica – Ospedale Santa Maria Goretti**	**Yes**	**Yes**	**Yes**
**14**	**UD - Ospedale S. Maria della Misericordia**	**Yes**	**Yes**	**Yes**
**15**	**PD - AULSS6 Euganea Alta Padovana Cittadella Camposanpiero**	**Yes**	**Yes**	**Yes**
**16**	**NA - Università degli Studi della Campania "Luigi Vanvitelli"**	**Yes**	**Yes**	**Yes**
**17**	**NA - P.O. Ospedale del Mare**	**Yes**	**Yes**	**Yes**
**18**	**CO - Ospedale Valduce**	**Yes**	**Yes**	**Yes**
**19**	**PN - Aviano CRO**	**Yes**	**Yes**	**Yes**
**20**	**AQ - Ospedale San Salvatore**	**Yes**	**Yes**	**Yes**
**21**	**VR - AOU Integrata Verona**	**Yes**	**Yes**	**Yes**
**22**	**PR - A.O.U. di Parma**	**Yes**	**Yes**	**Yes**
**23**	**MI - Istituto Europeo Oncologico IEO**	**Yes**	**Yes**	**Yes**
**24**	**BG - ASST Papa Giovanni XXIII**	**Yes**	**Yes**	**Yes**
**25**	**BS - Fondazione Poliambulanza**	**Yes**	**Yes**	**Yes**
**26**	**MI - IRCCS Ospedale San Raffaele**	**Yes**	**Yes**	**Yes**
**27**	**BA - IRCCS De Bellis - Castellana Grotte**	**Yes**	**Yes**	**Yes**
**28**	**NA - INT Pascale**	**Yes**	**Yes**	**Yes**
**29**	**LE - Ambulatorio Tumori Eredofamiliari-Oncologia Medica Ospedale “Vito Fazzi”**	**Yes**	**Yes**	**Yes**
**30**	**NA - AORN dei Colli Ospedale Monaldi**	**Yes**	**Yes**	**Yes**
**31**	**PA - Policlinico P. Giaccone e Laboratorio di Genetica Oncologica**	**Yes**	**Yes**	**Yes**
**32**	**PZ - Rionero in Volture - CROB**	**Yes**	**Yes**	**Yes**
**33**	**BR - P.O. “A.Perrino” ASL Brindisi**	**Yes**	**Yes**	**Yes**

**34**	**PA - Ospedale La Maddalena**	**Yes**	**Yes**	**Yes**
**35**	**FC - Istituto Scientifico Romagnolo (IRST) - Meldola**	**Yes**	**/**	**/**
**36**	**MO - Azienda Ospedaliero Universitaria Policlinico Modena**	**Yes**	**/**	**/**
**37**	**RM - AOU Policlinico Umberto I**	**Yes**	**/**	**/**
**38**	**SP - La Spezia ASL5 Liguria**	**Yes**	**/**	**/**
**39**	**RM - Istituto Nazionale Tumori "Regina Elena" (IFO)**	**Yes**	**/**	**/**
**40**	**VR - Legnago**	**Yes**	**/**	**/**

EC, Ethics Committee.
